# *Shigella sonnei*, an emerging multidrug-resistant sexually transmitted pathogen in Franche-Comté, France

**DOI:** 10.1080/22221751.2021.1969289

**Published:** 2021-08-22

**Authors:** Souheil Zayet, Timothée Klopfenstein, Alix Pierron, Pierre-Yves Royer, Lynda Toko, Pauline Garnier, Vincent Gendrin

**Affiliations:** a Department of Infectious Disease, Nord Franche-Comté Hospital, Trevenans, France; b Department of Microbiology, Nord Franche-Comté Hospital, Trevenans, France

**Keywords:** *Shigella sonnei*, sexually transmitted infections, multidrug-resistant, third-generation cephalosporins, shigellosis

## Abstract

*Shigella sonnei* (*S. sonnei*) is sometimes sexually transmitted. Men, who have sex with men (MSM), may have sexual behaviours different from heterosexual population, and thus may be at risk for *S. sonnei* infection. We describe three cases of multidrug-resistant *S. sonnei* in MSM (one HIV-infected patient and two patients receiving pre-exposure prophylaxis against HIV). *S. sonnei* was isolated from stool specimens and all patients were successfully treated with parenteral third-generation cephalosporins following laboratory confirmation that the isolates were resistant to azithromycin. Two men (patients 2 and 3) were linked epidemiologically. These cases highlight the emergence of this pathogen and its association with some sexual behaviours among MSM in Franche-Comté, France.

## Introduction

*Shigella*, a pathovar of *Escherichia coli*, is a Gram-negative bacterial pathogen, which causes bacillary dysentery or bloody diarrhoea in humans. This pathovar comprises four groups, *Shigella flexneri, Shigella sonnei, Shigella dysenteriae,* and *Shigella boydii*. Currently, *Shigella sonnei* (*S. sonnei*) is an emerging pathogen globally, and a common cause shigellosis predominant in high-income countries [1,2]. Usually, typical risk groups in shigellosis include paediatric population and travellers to developing countries [2]. In the last years, *S. sonnei* infections have significantly increased among men who have sex with men (MSM) [3–5], who are known to have different sexual behaviours and human immunodeficiency virus (HIV)-infected patients [3,5] and have been thought to be associated with sexual transmission. Because of widespread resistance to ampicillin (AMP) and trimethoprim-sulphamethoxazole (SMX-TMP), treatment with these drugs is not advised unless isolates are susceptible [6] and azithromycin (AZM) is considered as the first-line or second-line treatment for shigellosis [6].

Nowadays, treatment options for *S. sonnei* have become more limited due to the emerging multidrug-resistant *Shigella* isolates, particularly those with resistance to AZM and fluoroquinolones (FQ) [3,7].

We report three cases of sexually transmitted *S. sonnei* infections in MSM diagnosed in *Franche-Comté*, France: one patient treated with antiretroviral therapy (ART) for HIV infection and two receiving pre-exposure prophylaxis (PrEP) against HIV. The isolates were characterized by the same phenotypic antimicrobial susceptibility testing (AST).

## Method

We retrospectively studied three patients with *S. sonnei* infection in NFC (*Nord Franche-Comté*) Hospital, France. We collected demographic characteristics, comorbidities, and characteristics of the current infection and outcome, from patients’ medical records.

*S. sonnei* isolates were suspected by studying biochemical characteristics on Hektoen agar (bioMerieux, Marcy-l’Etoile, France) and Kligler-Hajna agar tube (Biorad, Marnes-la-Coquette, France) and identification as *Escherichia coli* by mass spectrometry MALDI-TOF (Microflex BRUKER, Bremen, Germany) since this technology is unable to distinguish these very similar species. Then, identification was confirmed by performing Api 20E strips (bioMerieux, Marcy-l’Etoile, France) and agglutination (Biorad, Marnes-la-Coquette, France).

Antibiotic susceptibility was tested by the disc diffusion assay (disc and Mueller Hinton agar: Biorad, Marnes-la-Coquette, France). Susceptibility to azithromycin was determined by measuring minimum inhibitory concentration (MIC) with E-test® strips (bioMerieux, Marcy-l'Etoile, France). CASFM/EUCAST breakpoints were used for the interpretation. Strains were sent to the *E. coli*, *Shigella*, *Salmonella* National Reference Center (CNR-ESS) in Paris, France and were identified through multi-locus sequence typing based on the core genome (cgMLST) analysis.

## Results

The patients’ mean age was 32 years [31–34] and all of them were MSM (one patient was receiving ART for HIV-infection and two patients were PrEP users). In our case series, all patients denied engaging in chemsex or using illicit drugs, and none of them travelled recently. All patients presented with fever and gastro-intestinal (GI) symptoms, especially diarrhoea; however, only one of the three patients was hospitalized. *S. sonnei* was isolated from stool sample. The complete serotyping by agglutination identified the same serotype of *S. sonnei g* in all patients*,* based on biochemical characteristics. In this case series, all strains of *S. sonnei* were resistant to AMP, AZM, FQ and SMX-TMP and only susceptible to third-generation cephalosporins. They all received antibiotic treatment based on ceftriaxone with favourable outcome.

### Case 1, July 2018

A 31-year-old male with no past history sought care for fever and GI symptoms such as nausea, diarrhoea and abdominal pain. He did not report any concurrent diarrhoeal disease in family members or friends, nor recent travels. This patient had multiple sexual contacts with passive anal and oral sex and reported that he had not recently used condoms. He did not smoke tobacco or electronic cigarettes, drink alcohol, nor use illicit drugs. He received PrEP against HIV consisting in tenofovir disoproxil fumarate 200 mg/emtricitabine 245 mg (TDF/FTC). Physical examination on admission revealed high body temperature of 40°C, tachycardia (110 beats/minute) with a diffuse abdominal tenderness. Laboratory tests showed hypokalaemia at 2.8 mmol/l with normal renal and liver functions and an elevated C-reactive protein (CRP) at 293 mg/l. Peripheral blood cultures were negative. Serology for Hepatitis C, HIV and syphilis was also negative and the patient was vaccinated against hepatitis A and B. Abdominal computed tomography (CT) showed right colitis with mesenteric lymphadenitis. Direct examination of stool sample revealed gram-negative bacilli and the culture isolated *S. sonnei.* The patient was admitted to the infectious diseases department and started on empiric antibiotics (azithromycin 500 mg daily orally) and support treatment. Susceptibility testing of the organism revealed a MIC > 256 mg/l for azithromycin (R) (Table I). Thus, antibiotics were modified to daily 1 g intravenous ceftriaxone for 7 days with a favourable outcome at discharge.

**Table 1.** Demographic, clinical characteristics, laboratory and imaging findings in MSM patients with Shigella sonnei infections, Nord Franche-Comte Hospital, France.

		Patient 1	Patient 2	Patient 3
*Demographic and socio-behavioural characteristics*				
Age, y		31	31	34
Recent travel		No	No	No
Sexual behaviour		MSM (genito-oral and digital-anal activities)	MSM (genito-oral activities)	MSM (genito-oral activities)
Chemsex		No	No	No
PrEP user		Yes (intermittent)	No, under ART	Yes (continuous)
HIV status/ (if positive: CD4/mm^3^)		Negative	Positive (984/mm^3^)	Negative
*Clinical characteristics, laboratory and imaging findings*				
Clinical presentation		Fever, nausea, diarrhoea, abdominal pain	Fever, chills, fatigue, diarrhoea, abdominal pain	Fever, diarrhoea
Duration of symptoms, days		7	5	2
Laboratory data (On admission)	White-cell count/mm^3^ (4,000–10,000/mm^3^)	17,910	8940	–
	Lymphocytes/mm^3^ (1500–4000/mm^3^)	930	2280	–
	Haemoglobin, g/dL (13.5–17.5 g/dL)	12.6	13.8	–
	Creatinine, μmol/L (65–120 μmol/L)	75	83	–
	Alanine aminotransferase, U/L (8–45 U/L)	14	17	–
	Aspartate aminotransferase, U/L (10–40 U/L)	33	35	–
	C-reactive protein, mg/L (<5 mg/L)	293	45	–
Radiological data	Abdominal imaging features	Colitis with mesenteric lymphadenitis	ND	ND
Serotyping and biotype determination*		ND	*Shigella sonnei g*	*Shigella sonnei g*
Antimicrobial Testing Susceptibility (MICs of *Shigella sonnei* isolates)				
Ampicillin		R	R	R
Amoxicillin-clavulanic acid		S	S	S
Cefotaxime/Ceftriaxone		S	S	S
Piperacillin-tazobactam		S	S	S
Imipenem/Ertapenem		S	S	S
Amikacin		S		
Azithromycin		R (>256 mg/l)	R (>256 mg/l)	R (>256 mg/l)
Ciprofloxacin		R	R	R
Trimethoprim/sulphamethoxazole		R	R	R

*S. sonnei g is non-specific antibody, which was not available to provide the infection source.

MSM: Men who have sex with men; PrEP: pre-exposure prophylaxis; HIV: human immunodeficiency virus; ART: antiretroviral therapy; ND: not determined; MICs: minimal inhibitory concentrations; R: resistant; S: susceptible.

### Case 2, October 2020

A 31-year-old male with Centers for Disease Control and Prevention (CDC) A1 stage of HIV infection presented with a 5-day history of fever, chills, fatigue and abdominal pain. His past medical history revealed several episodes of different sexually transmitted infections (STIs): a successfully treated secondary syphilis and recurrent genital ulcers caused by herpes simples virus type 1. He was currently treated with tenofovir disoproxil fumarate 200 mg/emtricitabine 245 mg/dolutegravir 50 mg (TDF/FTC/DTG) with a good compliance and undetectable HIV viral load. Physical examination was normal and the patient complained of watery bloody stools. *S. sonnei* was isolated in stool culture with the same antibiotic resistance profile to AMP, AZM, FQ and SMX-TMP. The complete serotyping by agglutination identified serotype *g*. The patient received a daily intramuscular (IM) injection of 1 g ceftriaxone for 7 days. All symptoms disappeared after 2 days, with no evidence of relapse afterwards.

### Case 3, October 2020

A 34-year-old MSM patient with no significant medical history, except continuous PrEP (TDF/FTC), presented with a 48-hour history of fever followed by mucousy diarrhoea. The patient was treated by his general practitioner with azithromycin and racecadotril for persistence of fluid diarrhoea. The stool culture grew the same pathogen (*S. sonnei*). The strain was identical (*S. sonnei g*) with the same susceptibility testing and MICs, as compared to case 2. The patient was treated with IM ceftriaxone (1 g per day for 5 days), which resulted in a rapid resolution of symptoms. The patient 3 reported having had unprotected sex with patient 2.

## Discussion

Sexually transmitted bowel and rectal diseases are common in MSM and are caused by a wide variety of infectious agents, such as *Campylobacter spp, Yersinia spp, Salmonella spp, Shigella spp*, with a recent large increase in the rate of male shigellosis cases associated with antimicrobial resistance.

*S. sonnei* is one of the bacteria that may be responsible for shigellosis in the general population and particularly in children as one of the major cause of bacillary dysentery in France [8], but is also known to be sexually transmitted with increased levels of antimicrobial resistance. In France, MSM-associated lineages have been isolated and defined within *Shigella flexneri* serotype 3a, *S. flexneri* serotype 2a and *S. sonnei*, with higher-than-normal levels of antimicrobial resistance. Baker et al. showed that four French isolates belonged to the major sub-lineage of *S. flexneri* 2a, and 13 and 10 French isolates clustered with sub-lineages 1 and 4 of *S. sonnei*, respectively, indicating the presence of these three sub-lineages in French MSM, since 2009 [9].

Several outbreaks of *S. sonnei* have already been reported worldwide in MSM [3–5] which may have different sexual behaviours, such as direct/indirect oro-genital intercourse and chemsex practice.

In our cases, all patients denied practising chemsex. Nevertheless, drug use is a known risk factor for STIs, by disinhibiting sexual behaviours, especially in HIV patients [10]. On the other hand, PrEP use for HIV may possibly influence sexual behaviours by reducing condom use, increasing STI prevalence [11] including emergence of *Shigella* infection in MSM [5].

Regarding antimicrobial resistance, *S. sonnei* has been found to adapt and equip with antimicrobial resistance genes more profoundly through mobile genetic elements as compared to other *Shigella* species [12]. With *S. sonnei* infections in MSM population, a high level of antibiotic resistance has appeared, especially concerning AZM [7] and CIP [3]*.* In a recent publication, Bourguignon et al. proved that there was a local dissemination of 27 drug-resistant *S. sonnei g* in *Bordeaux*, France (regarding serotyping, determination of biotype and cgMLST analysis) with high MICs for AZM (>256), such as our patients [13]. In China, some authors described an significant increase of antimicrobial resistance rate of *S. sonnei* to third-generation cephalosporin agents in isolates with the extended-spectrum beta-lactamases (ESBLs)-producing genes [14]. In addition to this, oral cephalosporin treatment for shigellosis should be avoided despite in vitro susceptibility, because of a demonstrated clinical failure [15].

Finally, we think that our data are relevant to clinical confirmation and microbiological evidence of *S. sonnei* infection with the same antimicrobial testing susceptibility and MICs for all three cases in MSM. However, there are a few limits of this study: (i) the number of patients is very limited; (ii) faecal-oral transmission seems to be excluded, based on anamnestic evidence, but cannot be completely ruled out; (iii) without the whole genome sequencing, it cannot be determined with certainty if the strains infecting our patients were from the same cluster.

To conclude, we describe three cases of multidrug-resistant *S. sonnei* infections in MSM in Franche-Comté, France. This case series raises concerns about the ability to manage the spread of resistant Shigellosis in this population, especially with alarming re-emergence of STIs. Parenteral third-generation cephalosporins should be an alternative option as an empiric treatment for dysentery in MSM, with possible IM administration in outpatients.

## Supplementary Material

Figure_Minor_R.jpeg

## References

[1] Shad AA, Shad WA. *Shigella sonnei*: virulence and antibiotic resistance. Arch Microbiol. 2021;203(1):45–58. 32929595 10.1007/s00203-020-02034-3 PMC7489455

[2] Cohen D, Korin H, Bassal R, et al. Burden and risk factors of *Shigella sonnei* shigellosis among children aged 0–59 months in hyperendemic communities in Israel. Int J Infect Dis. 2019;82:117–123. 30831222 10.1016/j.ijid.2019.02.031

[3] Chiou C-S, Izumiya H, Kawamura M, et al. The worldwide spread of ciprofloxacin-resistant *Shigella sonnei* among HIV-infected men who have sex with men, Taiwan. Clin Microbiol Infect. 2016;22(4):383.e11–383.e16. 10.1016/j.cmi.2015.12.021 26806133

[4] Morgan O, Crook P, Cheasty T, et al. *Shigella sonnei* outbreak among homosexual men, London. Emerg Infect Dis. 2006;12(9):1458–1460. 17073105 10.3201/eid1209.060282 PMC3298285

[5] Hinic V, Seth-Smith H, Stöckle M, et al. First report of sexually transmitted multi-drug resistant *Shigella sonnei* infections in Switzerland, investigated by whole genome sequencing. Swiss Med Wkly. 2018;148:w14645. 30141522 10.4414/smw.2018.14645

[6] Shane AL, Mody RK, Crump JA, et al. Infectious diseases society of America clinical practice guidelines for the diagnosis and management of infectious diarrhea. Clin Infect Dis. 2017;65(12):e45–e80. 29053792 10.1093/cid/cix669 PMC5850553

[7] Ingle DJ, Easton M, Valcanis M, et al. Co-circulation of multidrug-resistant Shigella among men who have sex with men in Australia. Clin Infect Dis. 2019;69(9):1535–1544. 30615105 10.1093/cid/ciz005

[8] Jones G, Lefèvre S, Donguy M-P, et al. Outbreak of shiga toxin-producing *Escherichia coli* (STEC) O26 paediatric haemolytic uraemic syndrome (HUS) cases associated with the consumption of soft raw cow’s milk cheeses, France, March to May 2019. Euro Surveill. 2018;9(1):1462. 10.2807/1560-7917.ES.2019.24.22.1900305 PMC6549459 31164190

[9] Baker KS, Dallman TJ, Field N, et al. Horizontal antimicrobial resistance transfer drives epidemics of multiple Shigella species. Nat Commun. 2018;9(1):1462. 29654279 10.1038/s41467-018-03949-8 PMC5899146

[10] González-Baeza A, Dolengevich-Segal H, Pérez-Valero I, et al. Sexualized drug use (chemsex) is associated with high-risk sexual behaviors and sexually transmitted infections in HIV-positive men who have sex with men: data from the U-SEX GESIDA 9416 study. AIDS Patient Care STDs. 2018;32(3):112–118. 29620925 10.1089/apc.2017.0263

[11] Jansen K, Steffen G, Potthoff A, et al. STI in times of PrEP: high prevalence of chlamydia, gonorrhea, and mycoplasma at different anatomic sites in men who have sex with men in Germany. BMC Infect Dis. 2020;20(1):110. 32033533 10.1186/s12879-020-4831-4 PMC7007644

[12] Muthuirulandi Sethuvel DP, Devanga Ragupathi NK, Anandan S, et al. Update on: Shigella new serogroups/serotypes and their antimicrobial resistance. Lett Appl Microbiol. 2017;64(1):8–18. 27783408 10.1111/lam.12690

[13] Bourguignon C. La Shigellose: une nouvelle IST?:92.

[14] Qian H, Liu G, Chen Y, et al. Increasing clinical resistance rate of *Shigella sonnei* to cefotaxime in Jiangsu province, China, between 2012 and 2015. Ann Transl Med. 2018;6(11):207. 30023370 10.21037/atm.2018.05.43 PMC6035990

[15] Collins JP, Friedman CR, Birhane MG, et al. Evidence of failure of oral third-generation cephalosporin treatment for *Shigella sonnei* infection. Open Forum Infect Dis [Internet]. 2020 Apr 1 [cited 2021 Mar 24];7(ofaa113). Available from: 10.1093/ofid/ofaa113. PMC7175969 32341933

