# Supplementary figures and images for: Shigella sonnei, an emerging multidrug-resistant sexually transmitted pathogen in Franche-Comté, France
Source: Emerg Microbes Infect. 2021 Aug 22;10(1):1702–5. doi: 10.1080/22221751.2021.1969289 (PMC8381955; doi:10.1080/22221751.2021.1969289)

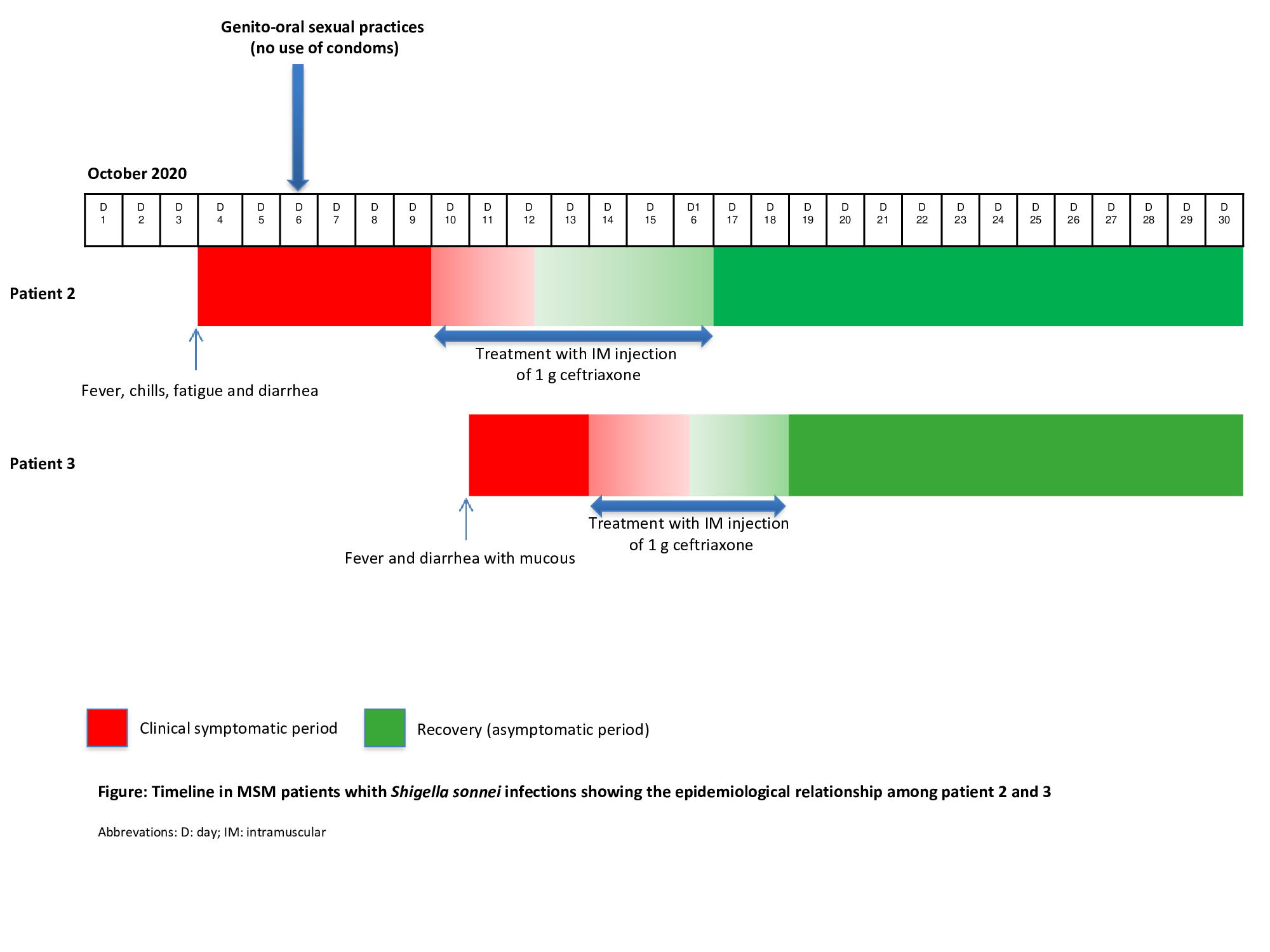

Supplement: Figure_Minor_R.jpeg [file TEMI_A_1969289_SM3596.jpeg]
